# Association of atopic dermatitis with autoimmune diseases: A bidirectional and multivariable two-sample mendelian randomization study

**DOI:** 10.3389/fimmu.2023.1132719

**Published:** 2023-03-30

**Authors:** Weixin Zhou, Jie Cai, Zifan Li, Ying Lin

**Affiliations:** ^1^ The Second Clinical College of Guangzhou University of Chinese Medicine, Guangzhou, China; ^2^ Department of Dermatology, The Second Affiliated Hospital of Guangzhou University of Chinese Medicine (Guangdong Provincial Hospital of Chinese Medicine), Guangzhou, China; ^3^ Guangdong Provincial Key Laboratory of Chinese Medicine for Prevention and Treatment of Refractory Chronic Diseases, Guangzhou, China

**Keywords:** atopic dermatitis, autoimmune diseases, mendelian randomization, asthma, causality

## Abstract

**Background:**

Observational studies have suggested the association between atopic dermatitis (AD) and the risks of autoimmune diseases. It is still unclear, however, whether or in which direction causal relationships exist, because these associations could be confounded.

**Objectives:**

Our study seeks to assess the possibility of AD as a cause of autoimmune diseases, and to estimate the magnitude of the causal effect.

**Methods:**

Two-sample mendelian randomization (MR) analyses were performed using genome-wide association study (GWAS) summary-level statistics. Specifically, bidirectional MR analyses were conducted to examine the direction of association of AD with autoimmune diseases; multivariable MR analyses (MVMR1) were used to test the independence of causal association of AD with autoimmune diseases after controlling other atopic disorders (asthma and allergic rhinitis), while MVMR2 analyses were conducted to account for potential confounding factors such as smoking, drinking, and obesity. Genetic instruments for AD (Ncases=22 474) were from the latest GWAS meta-analysis. The GWAS summary data for asthma and allergic rhinitis were obtained from UK Biobank. The GWAS summary data for smoking, alcohol consumption, obesity and autoimmune diseases (alopecia areata, vitiligo, systemic lupus erythematosus, ankylosing spondylitis, rheumatoid arthritis, and type 1 diabetes) were selected from the largest GWASs available. Causal estimates were derived by the inverse-variance weighted method and verified through a series of sensitivity analyses.

**Results:**

Genetically predicted AD linked to higher risks of rheumatoid arthritis (OR, 1.28; P=0.0068) (OR_MVMR1_, 1.65; P=0.0020) (OR_MVMR2_, 1.36; P<0.001), type 1 diabetes (OR, 1.37; P=0.0084) (OR_MVMR1_, 1.42; P=0.0155) (OR_MVMR2_, 1.45; P=0.002), and alopecia areata (OR, 1.98; P=0.0059) (OR_MVMR1_, 2.55; P<0.001) (OR_MVMR2_, 1.99; P=0.003) in both univariable and multivariable MR. These causal relationships were supported by sensitivity analyses. No causal effect of AD was identified in relation to systemic lupus erythematosus, vitiligo, and ankylosing spondylitis. Concerning the reverse directions, no significant association was noted.

**Conclusion:**

The results of this MR study provide evidence to support the idea that AD causes a greater risk of rheumatoid arthritis, type 1 diabetes and alopecia areata. Further replication in larger samples is needed to validate our findings, and experimental studies are needed to explore the underlying mechanisms of these causal effects.

## Introduction

Atopic dermatitis (AD) is a relapsing chronic skin condition affecting around 20% of children and 10% of adults in high-income countries (1). Symptoms of this condition include recurrent eczematous lesions, intense itching and discomfort, which can contribute to a severe negative impact on patient’s mental health, quality of life and working life (2, 3). Multiple factors interact to facilitate the development of AD, including genetic susceptibility, environmental factors, skin barrier dysfunction, altered microbiome, and immune dysregulation (4). Despite significant advances in the management of AD, a complete cure remains elusive (5). In recent years, research has increasingly shown an association between AD and non-atopic diseases such as infections, malignancies, and metabolic syndrome (6). Observational studies have further demonstrated that adults with AD are at a significantly higher risk of developing autoimmune diseases, particularly autoimmune cutaneous, gastrointestinal, and rheumatic diseases (7–9). However, to date, it remains unclear whether the association between AD and autoimmune disease is causal, as observational studies cannot establish causation due to potential confounding factors that were uncontrolled and unmeasured. Studying the cause-and-effect relationship between AD and autoimmune disease may lead to a deeper insight into the pathogenesis of AD, especially in the context of immune dysregulation. More reliable approaches are therefore needed for assessing causal relationships using observational data.

Mendelian randomization (MR) is a method of inferring exposure and outcome causality using genetic variants as instrumental variables (IVs). As genetic variants are single nucleotide polymorphisms (SNPs) independent of confounding factors or reverse causality (10). A recent MR study provided evidence proving that genetically predicted AD can increase the risk of autoimmune gastrointestinal diseases (11). Therefore, we implemented a bidirectional and multivariable two-sample MR analysis in this study to further investigate the causality between AD and autoimmune diseases of skin, connective tissues, and endocrine types, including alopecia areata (AA), vitiligo, systemic lupus erythematosus (SLE), ankylosing spondylitis (AS), rheumatoid arthritis (RA), and type 1 diabetes (T1D).

## Methods

### Study design

**Figure 1:**
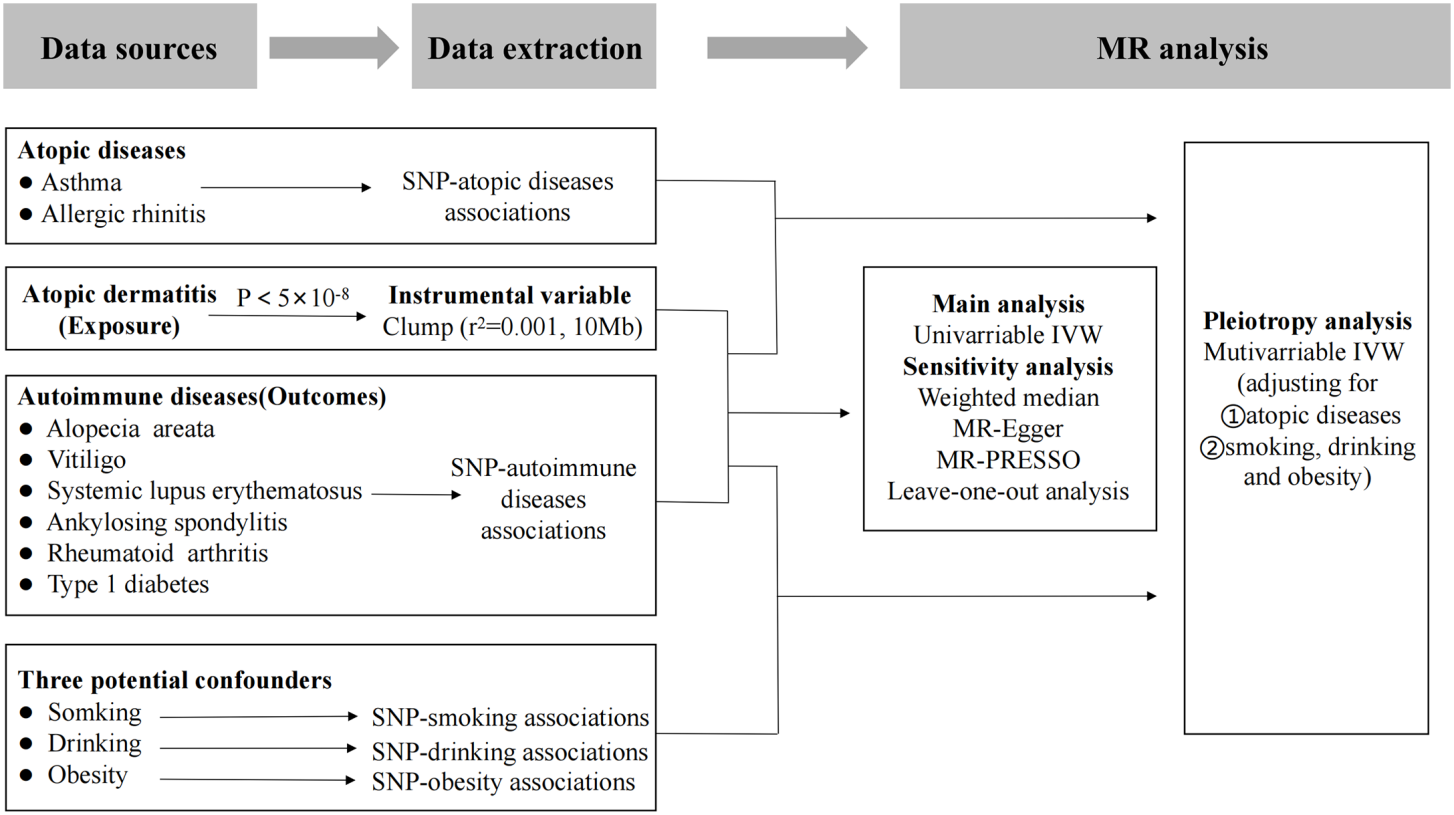
Flow chart of the study design and the process of MR analysis. MR, Mendelian randomization. MR-PRESSO, MR pleiotropy residual sum and outlier test. SNP, single nucleotide polymorphism. IVW, inverse-variance weighted.

To infer the direction of the causal relationship between AD and autoimmune diseases, bidirectional and multivariable two-sample MR analyses were performed using summary datasets of genome-wide association studies (GWASs). First, we performed univariable MR (UVMR) analyses to determine the causality between AD and autoimmune diseases in the forward direction. Second, we proceeded to identify the causality between autoimmune diseases and AD in the reverse direction. In addition, we conducted multivariable MR (MVMR) analyses on atopic disorders (AD, asthma, and allergic rhinitis) for autoimmune diseases to estimate whether AD is associated with autoimmune diseases independently. We also conducted further MVMR2 analyses involving atopic dermatitis, smoking, drinking, and obesity as exposures and six autoimmune diseases as outcomes. A detailed description of the multivariable and bidirectional two-sample MR design can be found elsewhere (12–14). Ethics approval and informed consent were not needed for this study, as it is based on publicly available summary-level GWAS data, and all original studies had already met these requirements. A flow chart illustrating the study design and the process of MR analysis is shown in **Figure 1**.

### Data sources

To carry out our Mendelian randomization (MR) analyses, we utilized summary-level data obtained from publicly available genome-wide association studies (GWAS) for each of the traits listed in **Table S1**. Specifically, we obtained genetic instrumental variables (IVs) for AD from the most recent and comprehensive meta-analysis study, which included a total of 796,661 individuals of European ancestry from the Estonian Biobank (11,187 cases and 125,537 controls), FinnGen (8,383 cases and 236,161 controls), and the UK Biobank (2,904 cases and 412,489 controls) (15). Genetic IVs for asthma (16) (56,167 cases and 352,255 controls), allergic rhinitis or hay fever (25,486 cases and 87,097 controls), and obesity (4,688 cases and 458,322 controls) were obtained from GWAS in the UK Biobank. Additionally, genetic IVs for RA (14,361 cases, 43,923 controls), T1D (9,266cases, 15,574 controls), SLE (5,201 cases, 9,066 controls), and vitiligo (4,680 cases, 39,586 controls) were obtained from three of the largest GWAS meta-analyses for each respective disease (17–20). Genetic IVs for AS (1,462 cases and 164,682 controls) and AA (289 cases and 211,139 controls) were both obtained from FinnGen (https://www.finngen.fi/en). Lastly, genetic IVs for smoking initiation (311,629 cases and 321,173 controls) and alcohol consumption (335,394 samples) were obtained from the GWAS and Sequencing Consortium of Alcohol and Nicotine use (21). Notably, all cases and controls in these studies were of European ancestry. There was minimal overlap between the GWAS populations used for exposure and outcome analyses, except for AD-AA and AD-AS, which had an overlap of approximately 26.54% and 20.86%, respectively.

### Genetic instrumental variable selection

First, with the exception of AA, we derived SNPs related to each trait with a significant threshold of p=5×10^−8^ from the full summary-level GWAS statistics. For AA, we selected SNPs on a lower significant threshold (p<5×10^−6^) owing to no SNP identified at p<5×10^−8^ in the AA summary GWAS statistics. Then, IVs were clumped within a genetic window of 10 Mb using a strict linkage disequilibrium (LD) threshold of r^2^ = 0.001 to determine that SNPs were independent. In the next step, we harmonized the effect estimates for both exposure and outcome variants, and excluded any possible SNPs with incompatible alleles or palindromic SNPs. For consistency, only SNPs available for all examined traits were used as IVs, and proxies were not used to replace those that were missing in outcome data. Further, F statistics (beta^2^/se^2^) (22)were used to assess the strength of genetically determined IVs (F>10, this is in line with the first MR assumption and not showing a bias towards weak IVs) (10, 23). **Table S2** displays details of the IVs that are ultimately used.

### Mendelian randomization analyses

For the main analysis, we applied the inverse-variance weighted (IVW) approach under a random-effects model, which permits heterogeneity across SNPs (24). In addition, we performed several sensitivity analyses to ensure the robustness of the main analysis. Weighted median (WM) method that requires over 50% of the weight corresponds to valid IVs was also applied to estimate the causal effects (25). MR-Egger intercepts were used to evaluate possible directional pleiotropy (26). MR-PRESSO framework was used to detect any possible horizontal pleiotropic outliers and correct the IVW estimate *via* outlier removal (27). The leave-one-out analysis was carried out to check whether the effect estimates were affected by a sole outlier variant.

Considering the strongest genetic correlation observed among atopic diseases, namely atopic dermatitis, asthma, and allergic rhinitis, as well as their similar associations with autoimmune diseases in observational studies (28–33), we undertook multivariable Mendelian randomization analyses (MVMR1) to examine the potential causal links between these three atopic conditions and six autoimmune diseases. **Table S3** provides a detailed breakdown of the instrumental variables employed in these analyses. To account for additional confounding factors such as smoking, drinking, and obesity, which may increase the risk of autoimmune diseases (34–39), we also conducted further MVMR2 analyses involving atopic dermatitis, smoking, drinking, and obesity as exposures and six autoimmune diseases as outcomes.

### Statistical analysis

We employed the statistical software R (version 4.1.2) and used the TwoSampleMR (version 0.5.6) and MR-PRESSO (version 1.0) packages for all analyses. For multiple testing, the evidential threshold (p<0.05) was corrected according to the number of exposures in each phase of analysis using the Bonferroni method [p<0.007 for bidirectional MR analyses (7 exposures); p<0.016 for MVMR1 analyses (3 exposures); p<0.0125 for MVMR2 analyses (4 exposures)]. Results with p<0.05 but not significant after Bonferroni adjustment were considered suggestive of an association.

## Results

### Associations of atopic dermatitis with autoimmune diseases

**Figure 2:**
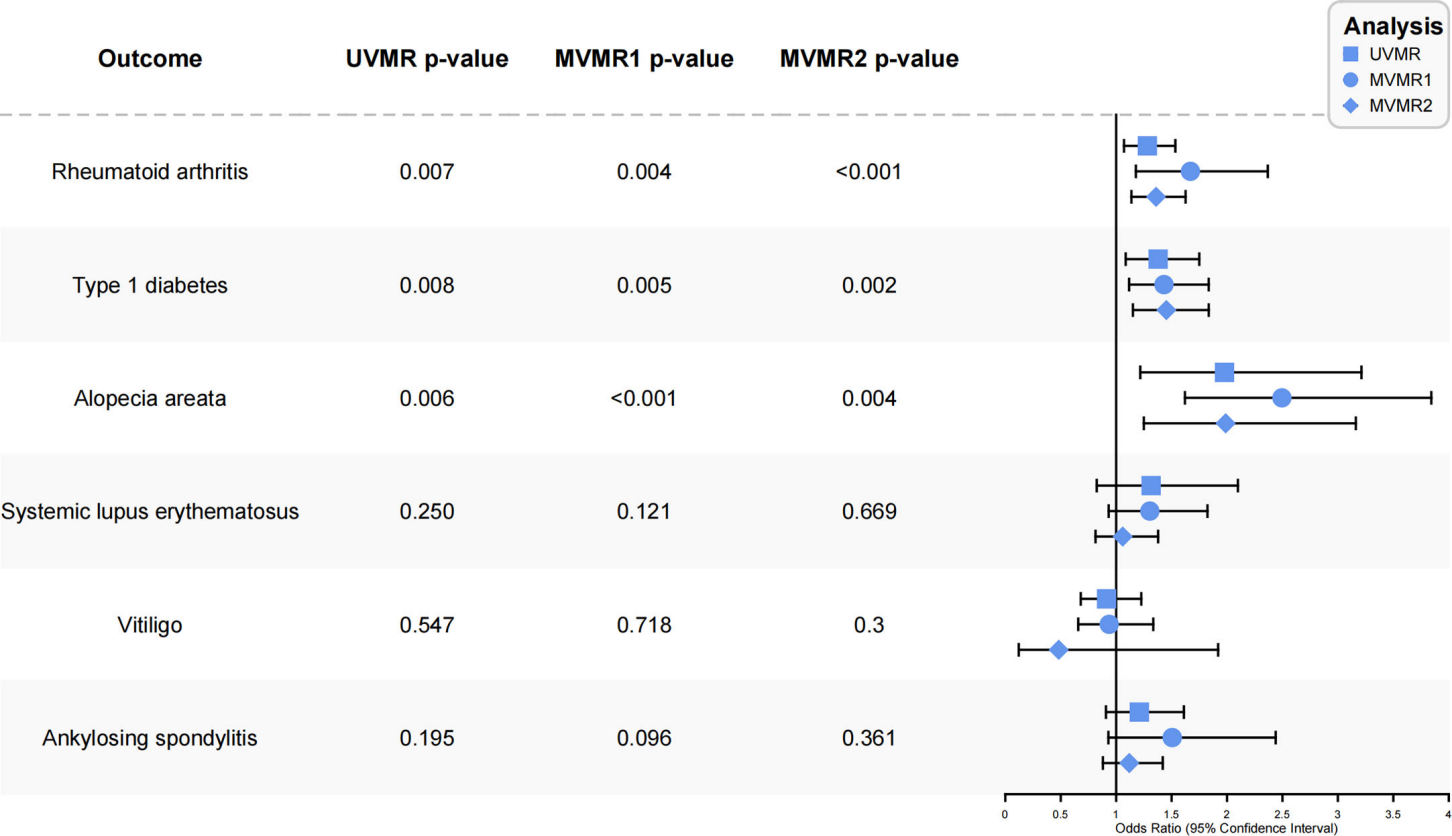
Forest plot of the causal association between atopic dermatitis and autoimmune diseases. UVMR, univariable mendelian randomization; MVMR, multivariable mendelian randomization; OR, odds ratio.

The final IVs used for univariable and multivariable MR analyses of AD and autoimmune disorders are presented in **Tables S2**, **S3**, respectively. Of note, all the SNPs (F-statistics of SNPs greater than 10) were strong instruments in UVMR analyses (**Table S2**). As shown in **Figure 2**, the UVMR analysis yielded a result that genetically predicted AD was significantly associated with an increased incidence of RA, adding 28% to the risk (IVW OR, 1.28; 95% CI, 1.07-1.53; p=0.0068). Similarly, when tested with MVMR analysis, there was a significant increase in the effect estimate for the risk of RA associated with AD (IVW OR_MVMR1_, 1.65; 95% CI, 1.20-2.27; P=0.0020) (IVW OR_MVMR2_, 1.36; 95% CI, 1.14-1.63; P<0.001). AD was also linked causally to a significantly higher risk of AA in the UVMR analysis (IVW OR, 1.98; 95% CI, 1.22-3.21; P=0.0059). Again, the causal estimates were consistent with the MVMR analysis (IVW OR_MVMR1_, 2.55; 95% CI, 1.65-3.93; P<0.001) (IVW OR_MVMR2_, 1.99; 95% CI, 1.24-3.16; P=0.003). We additionally observe a suggestive association between AD and T1D in UVMR analysis (IVW OR, 1.37; 95% CI, 1.09-1.75; P=0.0084). Surprisingly, AD was found to be causally associated with a significantly higher risk of T1D in the MVMR analysis (IVW OR_MVMR1_, 1.42; 95% CI, 1.07-1.88; P=0.0155) (IVW OR_MVMR2_, 1.45; 95% CI, 1.15-1.83; P=0.002). There was no significant indication of causal effects of AD on other autoimmune disorders (SLE, AS and vitiligo), either in UVMR or MVMR analyses. The detailed results of UVMR and MVMR analyses was shown in **Tables S4**–**S6**.

### Associations of autoimmune diseases with atopic dermatitis

When autoimmune diseases were used as exposures to test for bidirectional associations, no significant genetically predicted associations were seen (**Table S4**). However, there was evidence of a suggestive and small potential effect of genetically predicted vitiligo on the risk of AD (IVW OR, 0.97; 95% CI, 0.94-0.99; P=0.01).

### Sensitivity analysis

With weighted median method, there is evidence that the estimates of causal association of AD with RA (WM OR, 1.11; 95% CI, 0.95-1.29; P=0.1890), T1D (WM OR, 1.18; 95% CI, 0.96-1.46; P=0.1195) and AA (WM OR, 2.16; 95% CI, 1.14-4.08; P=0.0175) are consistent in direction with IVW (**Table S4**). There was no significant deviation from zero for all analyses in the MR-Egger intercept test (all p>0.05), implying no horizontal pleiotropy (**Table S7**). The MR-PRESSO suggested that there was significant horizontal pleiotropy in some analyses, yet the causal estimates of AD with RA (Outlier Corrected IVW OR, 1.21; SD, 0.08; P=0.0256) and T1D (Outlier Corrected IVW OR, 1.19; SD, 0.07; P=0.0201) were retained after outlier-corrected analyses (**Table S8**). The leave-one-out analysis revealed that the effect estimates were not affected by a sole outlier variant (**Figures S1**, **S2**).

## Discussion

The principal outcomes of the present study demonstrate compelling proof in favor of a considerable causal link between AD predicted genetically and RA, T1D, and AA, while taking other confounding factors into account. No indication exists that autoimmune diseases have a causal influence on AD once corrections for multiple testing have been made.

In consistent with our findings, almost all previous observational studies found that the prevalence of AA was significantly higher in patients with AD (7, 9, 40–42). There were conflicting results for the association between AD and RA suggested by a systematic review in 2017 (8). However, in recent years, several studies have supported an increased risk of RA in AD. For example, a systematic review and meta-analysis of observational studies published in 2021 found that patients with AD had a significantly higher risk of developing RA than those without AD (OR = 1.30; 95% CI, 1.17-1.44; I2, 48%) (43). This positive association is further supported by a 2022 cohort study from the UK National Database (40). Notably, there have been intense debates about the correlation between AD and T1D. A 2020 case-control study found a weak positive association between AD and T1D (OR = 1.08; 95% CI, 1.03-1.14; p=0.003) (7), while a 2017 systematic review suggested that most articles agreed on a lower risk of developing type 1 diabetes in AD patients (8). However, a 2022 cohort study and a 2019 cross-sectional study reported no significant association between AD and T1D (9, 40). Besides, about the correlation of AD with SLE, MS and AS, the results are conflicting in previous studies. For instance, a cross-sectional study from the USA and a case-control study from the Swedish National Registry found that adults with AD were at a significantly increased risk of SLE, AS, and MS (7, 9). A case-control study of adult AD from the Danish National Registry found significant associations of AD with SLE and AS, but not MS (41). However, a 2022 cohort Study from the UK revealed no significant association of AD with AS, SLE or MS (40). In contrast to our findings, a previous systematic review and a meta-analysis found a bidirectional relationship between AD and vitiligo and proposed that vitiligo increased the prevalence of AD (22, 44).

Our study extends previous observational studies, showing a significant causal association of AD with RA, T1D and AA, but not vice versa; no significant causal association of AD with SLE, ME and vitiligo, or vice versa. Despite the lack of potential pathogenic mechanisms for AD associated with RA, T1D and AA, it is hypothesized that immune dysregulation and shared genetic variants may be the potential mechanisms.

RA is a chronic autoimmune disease characterized by inflammation of the synovial joints, leading to progressive joint damage and functional impairment (45). B cells play a central role in the pathogenesis of RA by producing autoantibodies such as rheumatoid factor (RF) and anti-cyclic citrullinated peptide (CCP) antibodies, as well as contributing to the formation of ectopic lymphoid structures within the synovium that perpetuate the inflammatory response (46). The underlying mechanisms linking AD to RA are not yet fully understood, but several potential pathways have been proposed. One possible mechanism is through the activation and differentiation of B cells. AD is characterized by an imbalance in the Th1/Th2 immune response, with a predominance of Th2 cytokines such as IL-4 and IL-13. These cytokines can promote B cell into plasma cells (4), which produce autoantibodies against citrullinated proteins, a hallmark of RA (47). Another possible mechanism is through the activation of Th17 cells. AD is also characterized by an increase in the number of Th17 cells, which produce pro-inflammatory cytokines such as IL-17A, IL-22, and TNF-α (48). These cytokines can activate synovial fibroblasts and osteoclasts, leading to the destruction of the bone and cartilage in the joints (48, 49). In addition, recent studies have shown that Th17 cells can interact with B cells and promote the production of autoantibodies in the synovium (50).

T1D is characterized by the selective destruction of insulin-producing beta cells in the pancreatic islets of Langerhans, which is caused by an autoimmune response directed against these cells (51). This autoimmune response is mediated primarily by autoreactive T cells, which recognize beta cell-specific antigens and become activated, leading to the release of pro-inflammatory cytokines and chemokines, recruitment of other immune cells, and ultimately beta cell destruction (52). While Tregs can have a suppressive effect on autoreactive T cells and prevent the development of autoimmune diseases, they may also exhibit functional defects in AD patients (53). These defects might limit their ability to effectively control immune responses, ultimately leading to the activation of autoreactive T cells that target pancreatic beta cells and contribute to the development of T1D (54, 55). In addition, heightened production of pro-inflammatory cytokines generated by Th17 cells has demonstrated a positive correlation with the emergence of type 1 diabetes (T1D) (56, 57).

AA is an autoimmune disorder characterized by patchy hair loss due to the immune system attacking hair follicles (58). In both AD and AA, there is a Th2-skewed immune response, characterized by increased levels of Th2 cytokines such as IL-4, IL-5, and IL-13. These cytokines may promote the infiltration of T cells into the skin and hair follicles, leading to inflammation and damage to hair follicles (59, 60). Additionally, the activation of Th17 cells in AD has been suggested to play a contributory role in the production of proinflammatory cytokines, which may in turn stimulate inflammation and hair loss (61, 62).

Additionally, there is evidence of shared genetic factors between AD and other autoimmune diseases. A GWAS study that analyzed data from 21,000 cases and 95,000 controls identified multiple susceptibility loci linking AD to autoimmune diseases such as RA and T1D (63). Another GWAS study found that the Th2 cytokine IL-13 was a susceptibility locus for AA, providing support for a genetic connection between AA and AD (64). As it is well established, the most notable genetic factor contributing to skin barrier dysfunction in AD is the mutation of the Filaggrin gene (FLG) (4). A recent study suggested that FLG mutations not only increased the incidence of AA in patients with a history of AD, but also led to a worsening of AA (65).

In summary, it is evident that the interplay between AD and RA, T1D, and AA is intricate and likely entails multiple immune pathways and cell types. Further investigation is imperative to comprehensively elucidate the mechanisms underlying the causative influence of AD on RA, T1D, and AA and to formulate efficacious treatments for these autoimmune ailments.

## Strengths and limitations

Our study has several key strengths, which enhance the validity and reliability of our findings. Firstly, to the best of our knowledge, this is the first study that has systematically investigated the causal relationship between AD and autoimmune diseases of the skin, connective tissue, and endocrine system through a two-sample MR analysis. This innovative approach allowed us to investigate the causal effects of AD on autoimmune diseases in a comprehensive and rigorous manner. Furthermore, our study employed a variety of advanced MR techniques, including bidirectional MR, MVMR, and comprehensive sensitivity analyses, to enhance the validity of our findings. Bidirectional MR allowed us to assess the direction of causality between AD and autoimmune diseases, while MVMR enabled us to explore the potential interactions between atopic diseases. Additionally, our comprehensive sensitivity analysis demonstrated the robustness of our methods and indicated that our findings were not influenced by pleiotropic effects.

As with many MR studies, our study has some limitations that need to be acknowledged. Firstly, the limited number of cases with AA may affect the generalizability of the findings. As such, further research is required with larger sample sizes to fully understand the relationship between AD and AA. Secondly, there is some overlap between the GWAS populations of AD and AA, as well as AD and AS, thus, larger GWAS studies that include different populations are needed to replicate these MR findings. Thirdly, our study only considered six autoimmune diseases, and there may be other autoimmune diseases associated with AD that were not included in our analysis. Finally, as all the GWAS populations were of European ancestry, there is a potential for stratification bias, and the findings may not be generalizable to other ethnic groups.

In conclusion, the findings of the MR study provide genetic evidence that supports a causal relationship between AD and autoimmune diseases such as RA, T1D, and AA. These findings have important clinical implications, as they allow for the identification of individuals with AD who may be at higher risk for developing autoimmune diseases, enabling early diagnosis and treatment. Clinicians must be mindful of the symptoms associated with joint pain, scalp conditions, and dietary changes in AD patients to aid in the early diagnosis and prevention of autoimmune diseases. Additionally, clinicians should consider the potential benefits of using targeted treatments for AD, such as baricitinib (JAK1/2 inhibitor) and dupilumab (anti-IL-4Rα), which have been shown to have potential benefits in treating moderate to severe AA (66–68).

## Data availability statement

The original contributions presented in the study are included in the article/**Supplementary Material**. Further inquiries can be directed to the corresponding author.

## Author contributions

WZ designed the study. WZ and JC collected the data and drafted the article. YL and ZL reviewed and revised the manuscript. All authors contributed to the article and approved the submitted version.
