# Supplementary material for: Association of atopic dermatitis with autoimmune diseases: A bidirectional and multivariable two-sample mendelian randomization study
Source: Front Immunol. 2023 Mar 30;14:1132719. doi: 10.3389/fimmu.2023.1132719 (PMC10098361; doi:10.3389/fimmu.2023.1132719)
Supplement: Supplementary file 1 [file DataSheet_1.pdf]

Supplementary Material

1 Supplementary Figures and Tables

1.1 Supplementary Figures

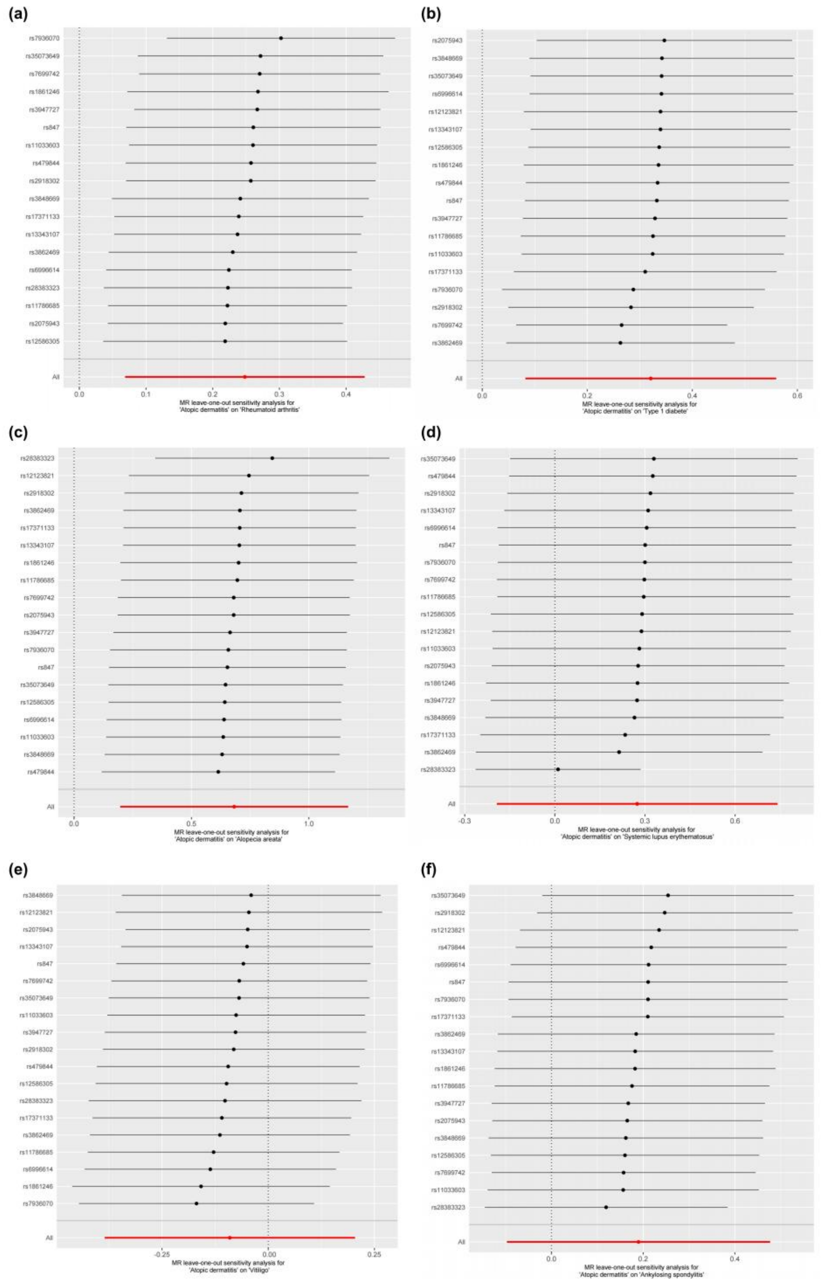

Supplementary Figure 1. MR leave-one-out sensitivity analyses for atopic dermatitis on autoimmune diseases

(a) MR leave-one-out sensitivity analysis for atopic dermatitis on rheumatoid arthritis

(b) MR leave-one-out sensitivity analysis for atopic dermatitis on type 1 diabetes

(c) MR leave-one-out sensitivity analysis for atopic dermatitis on alopecia areata

(d) MR leave-one-out sensitivity analysis for atopic dermatitis on systemic lupus erythematosus

(e) MR leave-one-out sensitivity analysis for atopic dermatitis on vitiligo

(f) MR leave-one-out sensitivity analysis for atopic dermatitis on ankylosing spondylitis

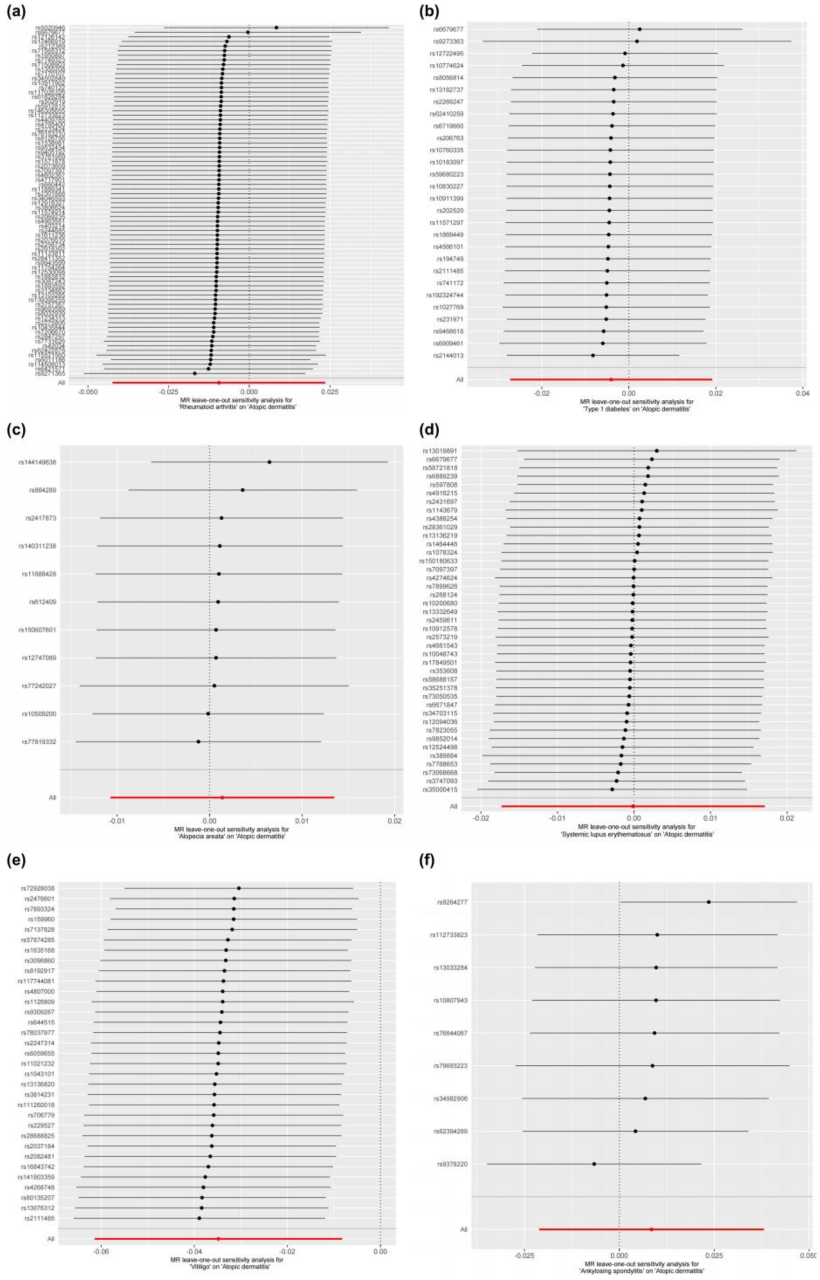

Supplementary Figure 2. MR leave-one-out sensitivity analyses for autoimmune diseases on atopic dermatitis

(a) MR leave-one-out sensitivity analysis for rheumatoid arthritis on atopic dermatitis

(b) MR leave-one-out sensitivity analysis for type 1 diabetes on atopic dermatitis

(c) MR leave-one-out sensitivity analysis for alopecia areata on atopic dermatitis

(d) MR leave-one-out sensitivity analysis for systemic lupus erythematosus on atopic dermatitis

(e) MR leave-one-out sensitivity analysis for vitiligo on atopic dermatitis

(f) MR leave-one-out sensitivity analysis for ankylosing spondylitis on atopic dermatitis

1.2 Supplementary Tables

Table S1. Data sources used to identify genetic variants in this study

Phenotype	Data source	Year	Category	Cases	Controls	Sample size	IEU ID
Atopic dermatitis	Sliz E et al.[15]	2021	Binary	22,474	774,187		-
Asthma	Valette K et al.[16]	2021	Binary	56,167	352,255		ebi-a-GCST90014325
Allergic rhinitis or Hayfever	UK Biobank	2018	Binary	25,486	87,097		ukb-b-7178
Smoking initiation	Liu M et al.[21]	2019	Binary	311,629	321,173	607,291	ieu-b-4877
Alcoholic drinks per week	Liu M et al.[21]	2019	Continuous	-	-	335,394	ieu-b-73
Obesity	UK Biobank	2018	Binary	4,688	458,322	463,010	ukb-b-15541
Rheumatoid arthritis	Okada Y et al.[17]	2014	Binary	14,361	43,923		ieu-a-832
Type 1 diabetes	Forgetta V et al.[20]	2020	Binary	9,266	15,574	24,840	ebi-a-GCST010681
Alopecia areata	FinnGen	2021	Binary	289	211,139		finn-b-L12_ALOPEC

						AREATA
Systemic lupus erythematosus	Bentham J et al.[18]	2015	Binary	5,201	9,066	ebi-a-GCST003156
Vitiligo	Jin Y et al.[19]	2016	Binary	4,680	39,586	-
Ankylosing spondylitis	FinnGen	2021	Binary	1,462	164,682	finn-b-M13_ANKYLOSPON

Table S2. The detailed information of the instrumental variables in each trait

Traits	SNP	P-value	EA	OA	Beta	SE	F
Atopic dermatitis	rs12123821*	2.20E-26	T	C	0.2704	0.0254	113.3302747
	rs17371133	2.07E-10	A	C	-0.0651	0.0102	40.73442907
	rs13343107	5.11E-10	A	G	-0.0754	0.0121	38.83040776
	rs1861246	1.52E-20	T	C	0.1134	0.0122	86.39854878
	rs7699742	5.45E-09	T	C	-0.0603	0.0103	34.27363559
	rs847	3.75E-17	T	C	0.0933	0.0111	70.65084003
	rs28383323*	1.10E-13	A	G	-0.1188	0.016	55.130625
	rs11786685	1.99E-11	A	G	0.0721	0.0108	44.56798697
	rs6996614	1.55E-08	A	C	0.0805	0.0142	32.13772069
	rs11033603	1.33E-12	A	G	0.1645	0.0232	50.27543475
	rs479844	2.41E-12	A	G	-0.0719	0.0103	48.72853238
	rs7936070	7.19E-19	T	G	0.0905	0.0102	78.72212611

	rs3947727	1.09E-11	T	C	0.0714	0.0105	46.24
	rs12586305	7.92E-11	T	G	-0.091	0.014	42.25
	rs3862469	3.59E-12	T	C	-0.0759	0.0109	48.48758522
	rs35073649	2.04E-14	T	C	0.0785	0.0103	58.08511641
	rs2918302	5.10E-12	A	G	0.0915	0.0133	47.33026174
	rs3848669	2.84E-15	T	G	0.1009	0.0128	62.13873291
	rs2075943	2.09E-09	A	G	0.0648	0.0108	36
Rheumatoid arthritis	rs12126142	1.01E-10	A	G	-0.0751	0.0116	41.91446195
	rs3761959	9.64E-11	T	C	0.0744	0.0115	41.8552741
	rs61828284	6.33E-09	T	C	-0.2018	0.0348	33.62666799
	rs2258734	6.04E-14	A	G	-0.0921	0.0123	56.06722189
	rs28411352	1.66E-11	T	C	0.0914	0.0136	45.16630623
	rs2301888	3.75E-26	A	G	-0.1282	0.0121	112.2549006
	rs6679677	1.41E-145	A	C	0.591	0.023	660.2665406
	rs1234313	1.90E-09	G	A	0.0797	0.0133	35.90983097
	rs2076616	6.20E-11	G	A	-0.0885	0.0135	42.97530864
	rs10911902	2.36E-08	T	C	-0.0847	0.0152	31.05128982
	rs12466919	1.59E-11	T	C	0.1025	0.0152	45.47372749
	rs11123811	2.01E-18	C	T	-0.0995	0.0114	76.17920899
	rs11889341	4.32E-30	T	C	0.1466	0.0129	129.1482483

rs1355208	6.77E-12	G	A	0.0818	0.0119	47.25118283
rs3087243	3.32E-24	A	G	-0.1261	0.0124	103.4157778
rs3806624	3.94E-11	G	A	0.0863	0.0131	43.3989278
rs4602367	1.76E-10	G	A	0.075	0.0117	41.09138725
rs13103285	4.29E-14	T	C	0.0989	0.0131	56.99673679
rs34046593	7.17E-17	A	G	0.1422	0.017	69.9683045
rs403214	3.96E-10	G	A	-0.0914	0.0146	39.19103021
rs2918392	4.62E-08	C	T	0.0668	0.0122	29.98011287
rs7731626	1.94E-26	A	G	-0.1956	0.0184	113.0061437
rs244685	6.04E-10	G	T	-0.089	0.0144	38.19926698
rs115521560	1.29E-103	C	A	0.7878	0.0364	468.4132653
rs9271365	1.00E-200	G	T	0.4888	0.0128	1458.285156
rs62422878	3.57E-09	T	C	0.1037	0.0176	34.71619964
rs12530098	1.35E-11	T	C	0.1382	0.0204	45.89398308
rs1611236	4.54E-19	A	G	-0.1165	0.0131	79.08775712
rs71565312	1.11E-67	A	G	0.699	0.0402	302.3446202
rs212389	6.66E-13	A	G	0.1058	0.0147	51.80082373
rs112733823	3.82E-24	T	C	0.191	0.0188	103.2169534
rs139395255	8.68E-60	G	A	0.3833	0.0235	266.0369217
rs114508013	1.82E-28	A	G	0.488	0.0441	122.4510363

rs5020946	1.00E-200	T	G	0.6519	0.0169	1487.950737
rs2233424	6.49E-26	T	C	0.1964	0.0187	110.3061569
rs7749323	3.47E-29	A	G	0.2835	0.0253	125.5639832
rs9405192	9.26E-11	A	G	-0.089	0.0137	42.20256806
rs11754264	1.88E-12	C	T	-0.1359	0.0193	49.58202905
rs146305655	3.29E-22	A	G	-0.4379	0.0452	93.85837282
rs76153210	6.83E-15	T	C	0.1597	0.0205	60.68790006
rs1571878	4.13E-40	T	C	-0.1539	0.0116	176.0196938
rs117026326	2.45E-19	T	C	0.381	0.0424	80.74548327
rs42034	1.28E-08	G	A	0.0871	0.0153	32.40809091
rs740122	5.37E-09	A	G	-0.0782	0.0134	34.05680552
rs4717901	9.52E-13	C	A	0.249	0.0349	50.90352296
rs3757387	1.87E-19	C	T	0.1236	0.0137	81.3946401
rs9693589	1.50E-18	A	G	0.1127	0.0128	77.52252197
rs10435844	9.73E-11	T	G	-0.0784	0.0121	41.98183184
rs11574914	9.92E-15	A	G	0.1153	0.0149	59.88059096
rs3134883	1.98E-15	A	G	0.0991	0.0125	62.853184
rs502919	6.17E-10	C	T	0.0829	0.0134	38.27361328
rs7097397	1.42E-12	A	G	-0.0847	0.012	49.82006944
rs71508903	3.13E-25	T	C	0.1487	0.0143	108.130911

rs2275806	2.51E-09	A	G	-0.0725	0.0122	35.31476754
rs1538981	4.42E-09	T	C	0.0671	0.0114	34.64458295
rs6421571	5.57E-14	C	T	0.134	0.0178	56.67213736
rs9943599	2.70E-10	T	C	0.083	0.0131	40.14334829
rs34502849	1.07E-09	A	G	-0.0851	0.014	36.94903061
rs4409785	7.85E-09	C	T	0.0982	0.017	33.36761246
rs660442	1.11E-09	A	G	-0.1067	0.0175	37.17515102
rs4963581	3.75E-08	A	G	0.0856	0.0156	30.10913872
rs9532434	1.94E-19	C	T	0.114	0.0126	81.85941043
rs1950897	1.02E-13	T	C	0.1069	0.0144	55.11000193
rs7170107	6.11E-18	T	C	0.1366	0.0158	74.74587406
rs8032939	4.47E-24	C	T	0.1244	0.0123	102.289378
rs12918327	3.04E-08	T	C	0.0867	0.0157	30.49571991
rs7206670	4.14E-09	T	G	0.0701	0.0119	34.70100982
rs4795400	5.86E-10	T	C	0.0743	0.012	38.33673611
rs2847297	2.65E-14	G	A	0.0903	0.0119	57.58131488
rs6011186	3.19E-10	T	C	-0.1074	0.0171	39.44721453
rs1883832	1.13E-16	C	T	0.1052	0.0127	68.61578523
rs1893592	1.48E-13	C	A	-0.0976	0.0132	54.67033976
rs2073609	1.47E-08	C	T	0.1029	0.0182	31.96597633

	rs8126756	1.81E-09	C	T	-0.0823	0.0137	36.08764452
	rs2069235	1.69E-20	A	G	0.1296	0.014	85.69469388
	rs5912815	4.79E-09	G	T	-0.0787	0.0134	34.49370684
Type 1 diabetes	rs6679677	3.42E-79	A	C	0.6527	0.0346	355.8566023
	rs10911399	6.75E-09	G	A	-0.3707	0.064	33.54943604
	rs2269247	7.28E-09	T	C	0.1709	0.0295	33.5614019
	rs1869449	4.55E-11	A	G	0.1769	0.0269	43.24651401
	rs11571297	1.11E-16	C	T	-0.1964	0.0237	68.67304029
	rs10183097	1.82E-10	C	T	0.2053	0.0322	40.65052467
	rs192324744	1.36E-10	G	T	0.562	0.0875	41.25309388
	rs6719660	2.52E-08	G	A	0.2918	0.0524	31.0104452
	rs2111485	1.89E-10	G	A	0.1577	0.0248	40.43523998
	rs1027769	3.52E-10	T	G	-0.9962	0.1588	39.35428973
	rs62410259	1.02E-12	A	G	-0.3796	0.0533	50.72218917
	rs13182737	1.49E-08	A	G	0.1465	0.0259	31.994529
	rs9273363	1.00E-200	A	C	1.2786	0.0334	1465.468428
	rs6909461	3.06E-21	C	A	-0.314	0.0332	89.45057338
	rs9468618	7.53E-10	T	C	-0.3009	0.0489	37.86401445
	rs206763	2.93E-18	A	G	0.6792	0.0779	76.01870012
	rs2144013	1.76E-12	G	A	0.2234	0.0317	49.66469962

	rs10760335	2.43E-08	G	A	0.1357	0.0243	31.18510051
	rs12722495	1.27E-14	C	T	-0.3145	0.0408	59.41840278
	rs10830227	1.02E-11	A	G	0.1582	0.0233	46.10002026
	rs59680223	5.00E-10	T	C	0.6421	0.1032	38.71202996
	rs10774624	1.34E-25	A	G	-0.2556	0.0244	109.7342112
	rs194749	5.37E-09	C	T	-0.1638	0.0281	33.9793569
	rs4566101	6.23E-12	C	T	0.1755	0.0255	47.36678201
	rs741172	3.11E-15	T	C	-0.2034	0.0258	62.15305571
	rs231971	1.55E-09	G	A	0.2411	0.0399	36.51309351
	rs8056814	1.99E-10	A	G	0.2641	0.0415	40.49865583
	rs202520	7.97E-10	G	A	-0.1573	0.0256	37.75526428
Alopecia areata	rs12747089	1.81E-06	C	T	0.5839	0.1223	22.7941833
	rs140311238	3.24E-06	A	G	2.1019	0.4515	21.67247901
	rs11888428	3.75E-06	T	G	2.6852	0.5806	21.38943013
	rs77242027	5.00E-07	G	A	3.2694	0.6505	25.26047511
	rs884289	2.43E-06	G	A	0.612	0.1298	22.23071645
	rs10509200	2.29E-06	C	T	0.4788	0.1013	22.34032016
	rs612409	3.57E-06	G	A	-0.3859	0.0833	21.46147439
	rs77819332	3.73E-06	T	C	3.3421	0.7224	21.40343146
	rs2417873	3.41E-06	A	G	0.504	0.1085	21.57752341

	rs144149638	4.51E-06	T	C	1.8738	0.4085	21.04080481
	rs150607601	3.57E-06	T	C	1.5125	0.3263	21.48605183
Systemic lupus erythematosus	rs6679677	4.55E-13	A	C	0.336472	0.0464854	52.39196829
	rs4661543	9.40E-11	G	T	0.274437	0.0423755	41.94262961
	rs10912578	1.65E-15	G	A	-0.24686	0.0309918	63.44652606
	rs17849501	1.81E-59	T	C	0.81093	0.0498642	264.4776777
	rs6671847	6.64E-12	A	G	0.198851	0.0289651	47.13087409
	rs4916215	5.07E-11	T	C	0.223144	0.0339693	43.15163271
	rs12094036	1.37E-08	C	T	-0.328504	0.0578595	32.23531894
	rs13019891	1.65E-83	T	G	-0.562119	0.0290336	374.8476147
	rs2573219	1.13E-42	C	A	0.587787	0.0429292	187.4711151
	rs10200680	4.96E-09	T	C	-0.248461	0.0424835	34.20391984
	rs268124	8.60E-09	T	C	0.18633	0.0323703	33.13386659
	rs2459611	7.62E-09	T	C	0.261365	0.045245	33.36980543
	rs4274624	9.73E-66	T	C	-0.559616	0.0326791	293.2513845
	rs10048743	2.04E-08	T	G	-0.231112	0.0412056	31.45810134
	rs34703115	4.08E-09	C	T	-0.616186	0.104778	34.58465476
	rs1464446	2.79E-16	T	G	-0.328504	0.0401497	66.94477944
	rs9852014	2.26E-36	G	A	0.620577	0.0492727	158.6275546
	rs13136219	3.50E-10	T	C	-0.174353	0.027787	39.37091669

rs1078324	7.11E-20	A	C	-0.71335	0.0781665	83.28446466
rs4388254	3.71E-10	T	C	0.378436	0.0603977	39.25943866
rs2431697	2.60E-14	C	T	-0.223144	0.0292964	58.01521611
rs6889239	2.19E-18	C	T	0.277632	0.03174	76.51123259
rs389884	2.92E-102	G	A	0.928219	0.0432319	460.9908042
rs7768653	3.11E-12	T	C	-0.207014	0.0296891	48.6189269
rs12524498	2.48E-08	T	G	-0.673345	0.120793	31.0736124
rs58721818	3.38E-18	T	C	0.65752	0.0755941	75.6557835
rs150180633	2.66E-41	T	C	0.928219	0.0689573	181.1925808
rs28361029	3.27E-10	A	G	-0.385662	0.0613604	39.50365995
rs35000415	1.86E-45	T	C	0.587787	0.041539	200.2294151
rs7823055	1.64E-34	T	G	-0.350657	0.0286208	150.1071876
rs7899626	4.19E-08	T	C	0.182322	0.0332532	30.06154268
rs7097397	8.60E-11	A	G	-0.18633	0.0287118	42.11576829
rs58688157	2.97E-11	G	A	-0.223144	0.0335647	44.19823106
rs353608	2.93E-11	G	A	0.18633	0.0280198	44.22170731
rs73050535	9.11E-09	T	C	-0.71335	0.124134	33.02355559
rs597808	3.51E-08	G	A	-0.162519	0.0294736	30.40478329
rs1143679	5.03E-48	A	G	0.582216	0.0399866	212.0016865
rs13332649	5.43E-17	G	A	-0.314711	0.0375683	70.17473145

	rs35251378	3.61E-13	A	G	-0.235722	0.0324266	52.84420952
	rs73068668	4.40E-08	A	G	-0.314711	0.0574903	29.96640854
	rs3747093	2.88E-14	A	G	0.262364	0.0345055	57.8138428
vitiligo	rs2476601	2.21E-14	A	G	0.329303747	0.04	67.77559868
	rs78037977	1.86E-13	G	A	0.285178942	0.04	50.82939318
	rs159960	8.08E-13	A	G	0.207014169	0.03	47.61651814
	rs16843742	6.11E-09	C	T	-0.210721031	0.04	27.75209565
	rs9309267	5.46E-13	T	C	0.392042088	0.05	61.47879944
	rs57874285	4.56E-08	A	C	0.157003749	0.03	27.38908571
	rs2111485	2.69E-22	A	G	-0.287682072	0.03	91.95663868
	rs3096860	1.02E-08	C	T	0.173953307	0.03	33.62194784
	rs60135207	2.44E-14	T	G	-0.223143551	0.03	55.32560499
	rs141903359	6.89E-15	G	A	0.31481074	0.04	61.9411262
	rs2037184	4.24E-09	C	T	-0.174353387	0.03	33.77678179
	rs13076312	4.63E-22	T	C	0.277631737	0.03	85.64375685
	rs13136820	1.72E-11	C	T	0.198850859	0.03	43.93518225
	rs28688825	1.76E-38	G	A	0.457424847	0.04	130.7734317
	rs72928038	1.42E-11	A	G	0.2390169	0.04	35.70567419
	rs2247314	1.30E-13	C	T	-0.235722334	0.03	61.73890947
	rs117744081	1.62E-21	G	A	0.657520003	0.07	88.23113352

	rs706779	9.87E-25	C	T	-0.301105093	0.03	100.7380854
	rs7893324	4.61E-08	C	T	-0.235722334	0.04	34.72813658
	rs3814231	1.71E-08	T	C	-0.186329578	0.03	38.57634634
	rs1043101	2.54E-13	G	A	0.207014169	0.03	47.61651814
	rs1126809	5.02E-32	A	G	-0.415515444	0.03	191.8367602
	rs11021232	1.91E-21	C	T	0.322083499	0.03	115.2642005
	rs644515	1.15E-09	A	G	0.173953307	0.03	33.62194784
	rs7137828	1.18E-14	T	C	-0.223143551	0.03	55.32560499
	rs111260018	9.67E-09	A	G	0.570979547	0.1	32.60176426
	rs8192917	1.37E-10	C	T	0.207014169	0.03	47.61651814
	rs1635168	6.97E-13	A	C	0.357674444	0.05	51.17240323
	rs4268748	1.23E-20	C	T	-0.314710745	0.03	110.0476144
	rs4807000	1.58E-09	A	G	0.173953307	0.03	33.62194784
	rs2082481	3.27E-09	A	G	-0.210721031	0.03	49.33705893
	rs6059655	3.58E-13	A	G	-0.46203546	0.06	59.29910165
	rs229527	1.40E-24	A	C	0.292669614	0.03	95.17278104
Ankylosing spondylitis	rs13033284	9.67E-09	C	T	-0.2214	0.0386	32.89884292
	rs9264277	3.73E-31	C	T	0.5198	0.0448	134.62215
	rs34982906	3.82E-19	C	T	0.8211	0.0918	80.00308642
	rs62394289	1.90E-11	A	G	0.3738	0.0557	45.03687038

	rs79693223	3.36E-34	T	C	1.2953	0.1062	148.7618935
	rs9378220	1.97E-35	A	C	-0.6933	0.0558	154.373945
	rs112733823	1.43E-14	T	C	0.36	0.0468	59.17159763
	rs76644067	9.44E-15	A	G	0.7328	0.0946	60.00516697
	rs10807943	4.10E-12	C	T	-0.5628	0.0812	48.039239

Table S3. The detailed information of the instrumental variables used in MVMR1

exposure	SNP	P-value	EA	OA	Beta	SE	EAF
Atopic dermatitis	rs4480384	0.06897	A	G	-0.0197	0.0108	0.3356
Atopic dermatitis	rs12123821	2.20E-26	T	C	0.2704	0.0254	0.0448
Atopic dermatitis	rs61815559	1.12E-16	A	T	-0.3689	0.0445	0.9774
Atopic dermatitis	rs2988277	0.1031	T	C	-0.0178	0.0109	0.3461
Atopic dermatitis	rs6662018	1.64E-09	T	G	-0.0668	0.0111	0.3129
Atopic dermatitis	rs78556180	0.8573	T	G	-0.0025	0.0141	0.1489
Atopic dermatitis	rs4081545	1.47E-09	T	C	-0.066	0.0109	0.6876
Atopic dermatitis	rs2296618	0.38	A	G	0.0138	0.0157	0.8785
Atopic dermatitis	rs10178845	0.0004796	A	G	-0.0386	0.0111	0.3056
Atopic dermatitis	rs72823641	7.32E-07	A	T	-0.0677	0.0137	0.1505
Atopic dermatitis	rs7423358	0.0001882	T	C	-0.0449	0.012	0.2532
Atopic dermatitis	rs34290285	0.01045	A	G	-0.0298	0.0116	0.2509

Atopic dermatitis	rs9879150	0.9588	T	C	5.00E-04	0.0102	0.6022
Atopic dermatitis	rs35570272	0.004313	T	G	0.0297	0.0104	0.3888
Atopic dermatitis	rs7626218	0.4994	A	T	0.0071	0.0105	0.6096
Atopic dermatitis	rs13099273	0.5209	A	T	-0.0066	0.0102	0.4763
Atopic dermatitis	rs1684466	0.3481	A	G	-0.0101	0.0108	0.6309
Atopic dermatitis	rs5743618	0.004627	A	C	-0.0369	0.013	0.2014
Atopic dermatitis	rs72669169	3.27E-05	T	C	0.0452	0.0109	0.6974
Atopic dermatitis	rs16903574	1.20E-07	C	G	-0.0854	0.0161	0.9009
Atopic dermatitis	rs6871748	2.84E-05	T	C	0.0462	0.011	0.7048
Atopic dermatitis	rs4957317	0.003803	T	C	0.0302	0.0104	0.6454
Atopic dermatitis	rs7734635	0.007805	A	G	-0.0338	0.0127	0.8216
Atopic dermatitis	rs1837253	0.2946	T	C	-0.0123	0.0117	0.2611
Atopic dermatitis	rs10477741	0.305	T	G	-0.014	0.0136	0.844
Atopic dermatitis	rs848	4.01E-17	A	C	0.0929	0.011	0.2634
Atopic dermatitis	rs413214	0.1049	A	G	0.0171	0.0106	0.6302
Atopic dermatitis	rs76493820	0.1672	C	G	-0.0265	0.0192	0.9317
Atopic dermatitis	rs28752924	4.95E-11	T	C	0.0985	0.015	0.6331
Atopic dermatitis	rs9272226	1.57E-08	T	C	-0.0835	0.0148	0.5304
Atopic dermatitis	rs802731	0.0005815	C	G	-0.0391	0.0114	0.731
Atopic dermatitis	rs3827780	0.0005593	A	G	-0.0354	0.0103	0.5636

Atopic dermatitis	rs7770794	0.697	A	G	0.0043	0.011	0.3081
Atopic dermatitis	rs10486391	0.1492	A	G	0.0146	0.0101	0.5544
Atopic dermatitis	rs35621564	0.2564	A	G	0.0118	0.0104	0.607
Atopic dermatitis	rs1608555	0.08592	T	C	0.0186	0.0109	0.3374
Atopic dermatitis	rs4722758	0.08323	C	G	-0.0228	0.0132	0.8145
Atopic dermatitis	rs4739738	8.27E-10	A	G	-0.0665	0.0108	0.6598
Atopic dermatitis	rs6996614	1.55E-08	A	C	0.0805	0.0142	0.449
Atopic dermatitis	rs9297768	0.498	A	G	0.0074	0.0109	0.3266
Atopic dermatitis	rs992969	0.9593	A	G	6.00E-04	0.0123	0.2379
Atopic dermatitis	rs919826	0.4363	T	C	0.011	0.0141	0.5903
Atopic dermatitis	rs1930778	0.005938	A	C	0.0288	0.0105	0.3835
Atopic dermatitis	rs11816044	0.1468	A	G	-0.0165	0.0114	0.2947
Atopic dermatitis	rs2477923	0.123	T	C	0.0159	0.0103	0.5246
Atopic dermatitis	rs1444782	0.381	A	G	-0.0095	0.0108	0.3679
Atopic dermatitis	rs1870140	0.01062	A	G	0.0324	0.0127	0.18
Atopic dermatitis	rs12782153	0.02759	A	G	0.0225	0.0102	0.4303
Atopic dermatitis	rs11042902	0.9554	T	C	-6.00E-04	0.011	0.3111
Atopic dermatitis	rs11033603	1.33E-12	A	G	0.1645	0.0232	0.0419
Atopic dermatitis	rs479844	2.41E-12	A	G	-0.0719	0.0103	0.4275
Atopic dermatitis	rs7936312	7.86E-19	T	G	0.0904	0.0102	0.4523

Atopic dermatitis	rs11236813	4.91E-10	C	G	-0.0982	0.0158	0.1106
Atopic dermatitis	rs12365699	0.000158	A	G	-0.0497	0.0132	0.1705
Atopic dermatitis	rs1689510	6.15E-06	C	G	0.0491	0.0109	0.3278
Atopic dermatitis	rs3024971	0.002137	T	G	0.0645	0.021	0.9198
Atopic dermatitis	rs3947727	1.09E-11	T	C	0.0714	0.0105	0.5991
Atopic dermatitis	rs7961712	3.91E-07	A	G	0.0758	0.015	0.8569
Atopic dermatitis	rs11513729	0.007561	T	C	-0.0278	0.0104	0.4013
Atopic dermatitis	rs668622	0.0001444	A	G	-0.0387	0.0102	0.5561
Atopic dermatitis	rs981625	0.1819	C	G	-0.0297	0.0223	0.9415
Atopic dermatitis	rs912131	0.001495	A	G	-0.0342	0.0108	0.3185
Atopic dermatitis	rs12586305	7.92E-11	T	G	-0.091	0.014	0.8411
Atopic dermatitis	rs28498223	0.02093	T	C	0.0261	0.0113	0.2842
Atopic dermatitis	rs11071559	0.004892	T	C	-0.0382	0.0136	0.1469
Atopic dermatitis	rs56375023	0.005552	A	G	0.0322	0.0116	0.2479
Atopic dermatitis	rs4842921	0.129	A	G	-0.0161	0.0106	0.3644
Atopic dermatitis	rs35441874	2.26E-07	A	T	-0.0621	0.012	0.2364
Atopic dermatitis	rs3785356	0.3477	T	C	0.0107	0.0114	0.2821
Atopic dermatitis	rs71368508	0.3014	A	C	-0.0478	0.0462	0.0154
Atopic dermatitis	rs4795401	0.1831	A	G	0.0135	0.0101	0.4799
Atopic dermatitis	rs112267124	0.3028	A	G	0.0186	0.0181	0.1743

Atopic dermatitis	rs3985697	0.752	T	C	0.0039	0.0124	0.2098
Atopic dermatitis	rs12964116	0.6896	A	G	0.0132	0.0331	0.972
Atopic dermatitis	rs117552144	0.6731	T	C	0.0125	0.0296	0.0478
Atopic dermatitis	rs2918302	5.10E-12	A	G	0.0915	0.0133	0.1741
Atopic dermatitis	rs117710327	0.001095	A	C	-0.0621	0.019	0.0778
Atopic dermatitis	rs8125525	0.1102	T	C	-0.0187	0.0117	0.2556
Atopic dermatitis	rs3848669	2.84E-15	T	G	0.1009	0.0128	0.7789
Atopic dermatitis	rs11088309	0.7046	C	G	-0.0059	0.0155	0.8646
Atopic dermatitis	rs2075943	2.09E-09	A	G	0.0648	0.0108	0.5761
Asthma	rs61815559	1.81E-12	T	A	0.128649	0.0182518	0.0344492
Asthma	rs10178845	8.22E-18	A	G	-0.0616566	0.00717229	0.294552
Asthma	rs848	5.80E-30	C	A	-0.0963344	0.00847156	0.817872
Asthma	rs72823641	2.56E-53	A	T	-0.147841	0.00961818	0.13665
Asthma	rs35570272	2.91E-14	T	G	0.0509447	0.00670108	0.396277
Asthma	rs35621564	7.50E-11	G	A	-0.0443909	0.00681856	0.365249
Asthma	rs9297768	1.27E-05	G	A	-0.03024	0.0069281	0.66556
Asthma	rs1870140	3.96E-08	G	A	-0.049794	0.00906558	0.845758
Asthma	rs11816044	1.20E-11	A	G	-0.0473268	0.00698072	0.325637
Asthma	rs479844	7.70E-10	G	A	0.0403223	0.00655551	0.554743
Asthma	rs7936312	9.54E-38	T	G	0.0838325	0.00652802	0.476762

Asthma	rs3785356	3.85E-15	T	C	0.0563	0.00716317	0.29795
Asthma	rs11513729	0.00145191	T	C	-0.0214222	0.00672781	0.411069
Asthma	rs4795401	1.16E-55	G	A	-0.102554	0.00652498	0.510615
Asthma	rs4739738	1.86E-23	A	G	-0.067912	0.00680464	0.641572
Asthma	rs1444782	2.14E-47	A	G	-0.095445	0.00660017	0.423577
Asthma	rs34290285	1.38E-39	A	G	-0.0988716	0.00750984	0.255414
Asthma	rs7423358	1.83E-08	C	T	0.0430394	0.00764835	0.758113
Asthma	rs6662018	0.121557	G	T	0.0110581	0.00714225	0.695029
Asthma	rs28752924	0.123074	G	T	-0.0183105	0.0118745	0.241006
Asthma	rs76493820	8.21E-10	G	C	0.0863742	0.0140655	0.0579568
Asthma	rs4957317	0.0115755	T	C	0.0178468	0.0070685	0.691024
Asthma	rs919826	4.65E-08	C	T	-0.0357527	0.00654304	0.489954
Asthma	rs12782153	0.0215343	G	A	-0.0151318	0.0065834	0.570338
Asthma	rs3024971	4.67E-26	G	T	-0.112196	0.0106267	0.107142
Asthma	rs12365699	1.16E-09	A	G	-0.0535351	0.00879601	0.166687
Asthma	rs7961712	7.35E-10	A	G	0.0564852	0.00917211	0.849901
Asthma	rs117710327	3.84E-21	A	C	-0.126406	0.013395	0.0669584
Asthma	rs8125525	0.00696498	T	C	-0.0201048	0.00745031	0.262283
Asthma	rs2075943	0.0949402	A	G	0.0109668	0.00656733	0.541234
Asthma	rs413214	2.68E-12	A	G	0.0469332	0.00671099	0.616158

Asthma	rs4480384	2.52E-10	G	A	0.043097	0.00681273	0.643257
Asthma	rs9879150	0.00101714	C	T	-0.0224826	0.00684246	0.358879
Asthma	rs1684466	4.92E-16	A	G	-0.0568948	0.00701248	0.637709
Asthma	rs13099273	6.21E-16	T	A	-0.0535817	0.00662712	0.507649
Asthma	rs72669169	3.91E-12	C	T	-0.0498189	0.00717809	0.291422
Asthma	rs1689510	6.09E-16	C	G	0.0556484	0.00688071	0.340371
Asthma	rs11042902	6.17E-09	T	C	0.0413305	0.00711112	0.307493
Asthma	rs35441874	2.47E-27	A	T	-0.0824693	0.00761464	0.247084
Asthma	rs11071559	4.21E-18	T	C	-0.0846874	0.00976462	0.128023
Asthma	rs4842921	1.17E-09	A	G	-0.0407093	0.00669088	0.387483
Asthma	rs3985697	0.808161	C	T	0.00207621	0.00855115	0.817381
Asthma	rs2918302	0.000673519	A	G	0.0308203	0.00906442	0.152975
Asthma	rs10486391	5.13E-09	G	A	-0.0388573	0.00665024	0.410809
Asthma	rs1608555	3.14E-08	T	C	0.0377471	0.00682176	0.358532
Asthma	rs10477741	3.44E-14	G	T	0.074266	0.00979669	0.128434
Asthma	rs9272226	2.37E-21	T	C	-0.0897705	0.00946208	0.628207
Asthma	rs2477923	3.64E-08	C	T	-0.0361313	0.00656022	0.463296
Asthma	rs802731	6.54E-11	G	C	0.0479182	0.00733727	0.270854
Asthma	rs12123821	1.81E-21	T	C	0.145713	0.0153134	0.0481732
Asthma	rs78556180	3.99E-08	T	G	0.0506806	0.00922962	0.146826

Asthma	rs4081545	1.05E-07	C	T	0.0374437	0.00704181	0.31125
Asthma	rs7626218	5.96E-10	T	A	-0.0413534	0.00667906	0.395198
Asthma	rs992969	1.25E-56	G	A	-0.119909	0.0075617	0.74764
Asthma	rs11236813	0.000456752	C	G	-0.0383432	0.0109398	0.099508
Asthma	rs912131	4.04E-16	G	A	0.0580773	0.00713704	0.703887
Asthma	rs3947727	0.188841	T	C	0.00874187	0.00665279	0.602103
Asthma	rs668622	6.24E-05	A	G	-0.0263284	0.0065763	0.561954
Asthma	rs117552144	8.51E-09	T	C	0.079499	0.0138067	0.0667517
Asthma	rs112267124	2.55E-08	A	G	0.0428843	0.00769944	0.234223
Asthma	rs71368508	6.97E-06	A	C	-0.103452	0.0230168	0.0210754
Asthma	rs12964116	1.08E-09	G	A	0.107619	0.0176522	0.0354621
Asthma	rs16903574	5.31E-12	G	C	0.0865509	0.0125488	0.0765121
Asthma	rs7734635	2.48E-17	G	A	0.0767811	0.00906625	0.154263
Asthma	rs5743618	2.86E-17	A	C	-0.0664448	0.00786149	0.225707
Asthma	rs6871748	3.42E-09	C	T	-0.0432282	0.00731418	0.27639
Asthma	rs6996614	0.0401782	A	C	0.0134424	0.00655117	0.526682
Asthma	rs1930778	0.00136587	C	A	-0.0218029	0.00680963	0.644415
Asthma	rs2988277	3.49E-11	T	C	-0.0441186	0.00666025	0.398654
Asthma	rs2296618	1.30E-10	G	A	-0.0618798	0.0096286	0.135293
Asthma	rs1837253	1.49E-47	C	T	0.108356	0.00748003	0.739654

Asthma	rs4722758	7.21E-15	G	C	0.0634119	0.00814979	0.19958
Asthma	rs3827780	2.02E-08	A	G	-0.0368473	0.00656749	0.555099
Asthma	rs7770794	8.81E-09	A	G	0.0415551	0.0072243	0.285802
Asthma	rs981625	4.16E-08	G	C	0.0724725	0.0132157	0.06495
Asthma	rs12586305	0.011323	G	T	0.0223392	0.00882077	0.163365
Asthma	rs11033603	0.0112551	A	G	0.0493603	0.019474	0.0296905
Asthma	rs28498223	1.05E-11	T	C	0.0497316	0.00731458	0.280804
Asthma	rs56375023	3.18E-41	A	G	0.103455	0.00769319	0.237326
Asthma	rs3848669	8.96E-06	T	G	0.0335932	0.0075645	0.753678
Asthma	rs11088309	8.55E-11	G	C	0.0604575	0.00931455	0.142544
Allergic rhinitis or Hayfever	rs78556180	0.00189998	T	G	0.00778119	0.00250122	0.145817
Allergic rhinitis or Hayfever	rs10178845	1.90E-07	A	G	-0.0100725	0.0019355	0.291835
Allergic rhinitis or Hayfever	rs6996614	0.6	A	C	-0.000924013	0.00176657	0.524297
Allergic rhinitis or Hayfever	rs7770794	0.000700003	A	G	0.00661781	0.0019513	0.285798
Allergic rhinitis or Hayfever	rs16903574	0.00189998	G	C	0.0105275	0.00339318	0.076121
Allergic rhinitis or Hayfever	rs668622	3.60E-11	A	G	-0.0117632	0.00177677	0.561674
Allergic rhinitis or Hayfever	rs3985697	1.50E-08	C	T	-0.0130664	0.00230813	0.816208
Allergic rhinitis or Hayfever	rs4842921	0.016	A	G	-0.00434295	0.0018076	0.389477
Allergic rhinitis or Hayfever	rs11042902	0.91	T	C	0.00021107	0.00192327	0.307783
Allergic rhinitis or Hayfever	rs6871748	1.50E-19	C	T	-0.0178809	0.00197748	0.273667

Allergic rhinitis or Hayfever	rs1837253	3.00E-13	C	T	0.014625	0.00200502	0.740356
Allergic rhinitis or Hayfever	rs1608555	0.52	T	C	0.00118464	0.00183902	0.358803
Allergic rhinitis or Hayfever	rs28752924	0.33	C	T	0.00178421	0.0018213	0.447848
Allergic rhinitis or Hayfever	rs72823641	1.50E-26	A	T	-0.0271943	0.00255073	0.138171
Allergic rhinitis or Hayfever	rs6662018	0.12	G	T	0.00303668	0.00192685	0.694836
Allergic rhinitis or Hayfever	rs2296618	0.11	G	A	-0.00419311	0.00258666	0.136771
Allergic rhinitis or Hayfever	rs802731	0.00109999	G	C	0.00645544	0.00198331	0.269098
Allergic rhinitis or Hayfever	rs10486391	0.00990011	G	A	-0.00462199	0.001791	0.410799
Allergic rhinitis or Hayfever	rs35621564	0.000239999	G	A	-0.00676441	0.00184169	0.364683
Allergic rhinitis or Hayfever	rs7734635	1.40E-17	G	A	0.0207441	0.00242939	0.15677
Allergic rhinitis or Hayfever	rs12586305	0.00530005	G	T	0.00665387	0.00238505	0.163093
Allergic rhinitis or Hayfever	rs3848669	0.00830004	T	G	0.0053942	0.00204251	0.751952
Allergic rhinitis or Hayfever	rs10477741	0.016	G	T	0.00639991	0.00266153	0.12584
Allergic rhinitis or Hayfever	rs4739738	0.000109999	A	G	-0.00708932	0.00183563	0.642569
Allergic rhinitis or Hayfever	rs4795401	1.70E-05	G	A	-0.00758139	0.00176515	0.50732
Allergic rhinitis or Hayfever	rs2075943	0.36	A	G	-0.00162041	0.00176959	0.541242
Allergic rhinitis or Hayfever	rs1689510	5.70E-08	C	G	0.0101007	0.0018609	0.340493
Allergic rhinitis or Hayfever	rs5743618	3.90E-23	A	C	-0.0202992	0.00204903	0.238126
Allergic rhinitis or Hayfever	rs3947727	0.83	T	C	0.000372777	0.00178777	0.597588
Allergic rhinitis or Hayfever	rs11236813	0.0420001	C	G	-0.00595224	0.00293033	0.100938

Allergic rhinitis or Hayfever	rs28498223	7.80E-09	T	C	0.0113727	0.00197013	0.282331
Allergic rhinitis or Hayfever	rs912131	0.58	G	A	0.00105855	0.00192552	0.703026
Allergic rhinitis or Hayfever	rs13099273	0.0219999	T	A	-0.00408203	0.0017879	0.505702
Allergic rhinitis or Hayfever	rs12123821	0.00929994	T	C	0.0107858	0.00414996	0.04698
Allergic rhinitis or Hayfever	rs61815559	0.49	T	A	-0.00341482	0.00499241	0.033568
Allergic rhinitis or Hayfever	rs4722758	1.60E-05	G	C	0.00945315	0.0021879	0.201866
Allergic rhinitis or Hayfever	rs34290285	5.90E-19	A	G	-0.0179249	0.00201524	0.25825
Allergic rhinitis or Hayfever	rs479844	0.00269998	G	A	0.00530334	0.0017705	0.554608
Allergic rhinitis or Hayfever	rs413214	0.11	A	G	0.00293266	0.00181837	0.619375
Allergic rhinitis or Hayfever	rs848	0.12	C	A	-0.00357449	0.00226956	0.816185
Allergic rhinitis or Hayfever	rs1930778	6.70E-09	C	A	-0.0106639	0.00183903	0.6455
Allergic rhinitis or Hayfever	rs9297768	8.40E-11	G	A	-0.0121525	0.00187176	0.664938
Allergic rhinitis or Hayfever	rs2477923	0.0870001	C	T	-0.00303267	0.00177075	0.467129
Allergic rhinitis or Hayfever	rs981625	0.0015	G	C	0.011327	0.00357251	0.06471
Allergic rhinitis or Hayfever	rs56375023	2.20E-10	A	G	0.0131376	0.00207047	0.236813
Allergic rhinitis or Hayfever	rs1870140	0.0759994	G	A	-0.00433495	0.00244339	0.845648
Allergic rhinitis or Hayfever	rs2918302	0.000280001	A	G	0.00889007	0.00244471	0.153167
Allergic rhinitis or Hayfever	rs35441874	6.60E-12	A	T	-0.0140272	0.00204303	0.249619
Allergic rhinitis or Hayfever	rs112267124	0.48	A	G	-0.00146318	0.00207372	0.234846
Allergic rhinitis or Hayfever	rs4957317	1.10E-09	T	C	0.0116235	0.00190639	0.68843

Allergic rhinitis or Hayfever	rs72669169	1.80E-14	C	T	-0.0147499	0.00192467	0.295462
Allergic rhinitis or Hayfever	rs1684466	0.021	A	G	-0.00437997	0.00189134	0.638234
Allergic rhinitis or Hayfever	rs7423358	0.00700003	C	T	0.00557688	0.00206707	0.759471
Allergic rhinitis or Hayfever	rs3024971	4.50E-07	G	T	-0.0143675	0.00284732	0.10719
Allergic rhinitis or Hayfever	rs12964116	0.18	G	A	0.00635993	0.00469938	0.036432
Allergic rhinitis or Hayfever	rs35570272	0.032	T	G	0.00387565	0.00181165	0.39811
Allergic rhinitis or Hayfever	rs11816044	0.00359998	A	G	-0.00548431	0.00188326	0.32671
Allergic rhinitis or Hayfever	rs1444782	5.80E-06	A	G	-0.00807668	0.00178085	0.424853
Allergic rhinitis or Hayfever	rs919826	0.00449997	C	T	-0.0050253	0.0017707	0.493055
Allergic rhinitis or Hayfever	rs8125525	1.20E-10	T	C	-0.0130098	0.00201842	0.260782
Allergic rhinitis or Hayfever	rs11088309	0.0379997	G	C	0.00519844	0.00250768	0.143653
Allergic rhinitis or Hayfever	rs3785356	0.0129999	T	C	0.00479183	0.00193608	0.298648
Allergic rhinitis or Hayfever	rs12365699	2.00E-07	A	G	-0.012346	0.00237457	0.166536
Allergic rhinitis or Hayfever	rs12782153	1.60E-08	G	A	-0.010059	0.00177881	0.572927
Allergic rhinitis or Hayfever	rs11033603	0.77	A	G	0.00157161	0.00530654	0.02911
Allergic rhinitis or Hayfever	rs4081545	9.80E-06	C	T	0.00837951	0.00189519	0.314939
Allergic rhinitis or Hayfever	rs7936312	3.00E-19	T	G	0.0158265	0.00176432	0.476887
Allergic rhinitis or Hayfever	rs7626218	0.0025	T	A	-0.00544658	0.0017991	0.394886
Allergic rhinitis or Hayfever	rs11071559	8.20E-09	T	C	-0.015145	0.00262729	0.128794
Allergic rhinitis or Hayfever	rs7961712	0.2	A	G	0.00315493	0.00247332	0.849508

Allergic rhinitis or Hayfever	rs11513729	1.50E-10	T	C	-0.0115997	0.0018109	0.412436
Allergic rhinitis or Hayfever	rs9272226	0.37	T	C	-0.0015944	0.00178881	0.634679
Allergic rhinitis or Hayfever	rs3827780	0.15	A	G	-0.00256635	0.00176954	0.556987
Allergic rhinitis or Hayfever	rs992969	2.10E-10	G	A	-0.012859	0.00202416	0.746211
Allergic rhinitis or Hayfever	rs2988277	8.20E-05	T	C	-0.00710617	0.00180417	0.395088
Allergic rhinitis or Hayfever	rs4480384	0.017	G	A	0.00436267	0.00182708	0.633488
Allergic rhinitis or Hayfever	rs117710327	4.10E-06	A	C	-0.0165801	0.00359919	0.066877
Allergic rhinitis or Hayfever	rs71368508	2.80E-09	A	C	-0.0369408	0.0062136	0.020799
Allergic rhinitis or Hayfever	rs117552144	0.000409996	T	C	0.0133703	0.00378139	0.065043
Allergic rhinitis or Hayfever	rs9879150	2.80E-09	C	T	-0.0109859	0.00184902	0.356816
Allergic rhinitis or Hayfever	rs76493820	0.017	G	C	0.00909619	0.00381872	0.057531

SNP, single-nucleotide polymorphism; P-value is for the genetic association; EA, effect allele; OA, other allele; Beta, the per-allele effect on each trait; SE, standard error; EAF, effect allele frequency; F, F statistic.

Table S4. The results of MR analysis using inverse-variance weighted and weighted median methods

Exposure	Outcome	Method	nSNP	SE	OR	OR_LCI95	OR_UCI95	P-value
Atopic dermatitis	Rheumatoid arthritis	Inverse variance weighted	18	0.091622753	1.281704037	1.071018297	1.533834896	0.006752091
Atopic dermatitis	Rheumatoid arthritis	Weighted median	18	0.078199545	1.108184212	0.95070803	1.291744899	0.188981242
Atopic dermatitis	Type 1 diabetes	Inverse variance weighted	18	0.121745358	1.378324378	1.085724647	1.749778912	0.008399583

Atopic dermatitis	Type 1 diabetes	Weighted median	18	0.107837437	1.18281449	0.957466432	1.461200175	0.11948398
Atopic dermatitis	Alopecia areata	Inverse variance weighted	19	0.247594385	1.977316561	1.217081353	3.212423534	0.00589694
Atopic dermatitis	Alopecia areata	Weighted median	19	0.324047984	2.160044124	1.144530604	4.076597516	0.017473578
Atopic dermatitis	Systemic lupus erythematosus	Inverse variance weighted	19	0.23829035	1.315080238	0.824357153	2.097920819	0.2503794
Atopic dermatitis	Systemic lupus erythematosus	Weighted median	19	0.135983238	1.070749202	0.820231587	1.397780665	0.615176151
Atopic dermatitis	Vilitigo	Inverse variance weighted	19	0.150279474	0.913486793	0.680427413	1.226373461	0.547093892
Atopic dermatitis	Vilitigo	Weighted median	19	0.149416525	0.873793056	0.651962588	1.171101408	0.366566435
Atopic dermatitis	Ankylosing spondylitis	Inverse variance weighted	19	0.146319617	1.208939756	0.907517246	1.610476649	0.194708336
Atopic dermatitis	Ankylosing spondylitis	Weighted median	19	0.164589829	1.128879117	0.81760916	1.558651887	0.461408727
Rheumatoid arthritis	Atopic dermatitis	Inverse variance weighted	76	0.016813982	0.990623596	0.958509271	1.023813894	0.575284351
Rheumatoid arthritis	Atopic dermatitis	Weighted median	76	0.017328003	0.991954995	0.958831017	1.026223281	0.641104349
Type 1 diabetes	Atopic dermatitis	Inverse variance weighted	28	0.011839396	0.995950927	0.973105758	1.019332421	0.731828497
Type 1 diabetes	Atopic dermatitis	Weighted median	28	0.008888635	0.992054775	0.974921152	1.009489511	0.369487796
Alopecia areata	Atopic dermatitis	Inverse variance weighted	11	0.00616323	1.001378073	0.989354265	1.013548009	0.823191527

Alopecia areata	Atopic dermatitis	Weighted median	11	0.008246251	1.004626591	0.988519678	1.020995951	0.575643117
Systemic lupus erythematosus	Atopic dermatitis	Inverse variance weighted	41	0.00877253	0.999876137	0.982831065	1.017216818	0.988733985
Systemic lupus erythematosus	Atopic dermatitis	Weighted median	41	0.009551365	1.002481352	0.9838888	1.021425248	0.795273727
Vilitigo	Atopic dermatitis	Inverse variance weighted	33	0.013559548	0.965772314	0.940443331	0.991783484	0.010215117
Vilitigo	Atopic dermatitis	Weighted median	33	0.013387178	0.980558568	0.955164434	1.006627834	0.14249981
Ankylosing spondylitis	Atopic dermatitis	Inverse variance weighted	9	0.015098776	1.008522944	0.979114416	1.038814781	0.574056629
Ankylosing spondylitis	Atopic dermatitis	Weighted median	9	0.014928193	1.006428	0.977407295	1.036310374	0.667765576

MR, mendelian randomization; nSNP, number of single nucleotide polymorphism; SE, standard error; OR, odds ratio; CI, confidence interval; LCI, lower confidence interval; UCI, upper confidence interval

Table S5. The results of MVMR analysis 1

Exposure	Outcome	nSNP	SE	OR	OR_LCI95	OR_UCI95	P-value
Atopic dermatitis	Rheumatoid arthritis	16	0.161974621	1.649424888	1.200762073	2.26572984	0.002004684
Asthma	Rheumatoid arthritis	54	0.238370386	2.137568336	1.339723077	3.410554366	0.001437933
Allergic rhinitis or Hayfever	Rheumatoid arthritis	23	1.461223656	0.029410485	0.001677581	0.515609447	0.015807822
Atopic dermatitis	Type 1 diabetes	18	0.144477589	1.418505101	1.068683478	1.882836934	0.015530021

Asthma	Type 1 diabetes	59	0.218113798	1.574113559	1.026535202	2.41378327	0.037518975
Allergic rhinitis or Hayfever	Type 1 diabetes	23	1.342539606	0.097995452	0.007053637	1.36144062	0.083598249
Atopic dermatitis	Alopecia areata	18	0.221924726	2.546029884	1.648000564	3.933413804	2.54E-05
Asthma	Alopecia areata	59	0.329632947	1.47815223	0.774693539	2.820384971	0.235804284
Allergic rhinitis or Hayfever	Alopecia areata	23	2.032743967	0.023158905	0.000430935	1.244585157	0.063973983
Atopic dermatitis	Systemic lupus erythematosus	16	0.168854274	1.282450721	0.921104759	1.785551357	0.140670366
Asthma	Systemic lupus erythematosus	45	0.241070329	1.409986663	0.879046396	2.261612581	0.15409123
Allergic rhinitis or Hayfever	Systemic lupus erythematosus	19	1.469676357	0.182017944	0.010211756	3.244352061	0.246374347
Atopic dermatitis	Vitiligo	15	0.18138771	0.965687064	0.676762175	1.377960441	0.84735785
Asthma	Vitiligo	54	0.268294729	1.47915454	0.874251662	2.502595361	0.144535711
Allergic rhinitis or Hayfever	Vitiligo	21	1.653382899	0.628184678	0.024586652	16.05000884	0.778561731
Atopic dermatitis	Ankylosing spondylitis	18	0.22984236	1.537504179	0.979874686	2.412470834	0.061269507
Asthma	Ankylosing spondylitis	59	0.340932069	0.918098994	0.470632891	1.791004792	0.802095076
Allergic rhinitis or Hayfever	Ankylosing spondylitis	23	2.101398259	1.606085734	0.026122962	98.74498172	0.8216145

Table S6. The results of MVMR analysis 2

Exposure	Outcome	nSNP	SE	OR	OR_LCI95	OR_UCI95	P-value
Atopic dermatitis	Rheumatoid arthritis	13	0.091101457	1.359938384	1.137554221	1.625797149	0.000738982
Alcoholic drinks per week	Rheumatoid arthritis	15	0.4856155	1.071770913	0.413749715	2.776298927	0.88650271
Smoking initiation	Rheumatoid arthritis	54	0.180917422	1.069345337	0.75009792	1.524466896	0.710941124
Obesity	Rheumatoid arthritis	1	10.20034431	0.000670661	1.3925E-12	323006.8411	0.473761376
Atopic dermatitis	Type 1 diabetes	17	0.119052906	1.453025436	1.150623758	1.834902939	0.001698133
Alcoholic drinks per week	Type 1 diabetes	23	0.510431058	0.51328222	0.188741991	1.395866579	0.191348445
Smoking initiation	Type 1 diabetes	75	0.203926415	1.134716018	0.76085433	1.692282466	0.535425557
Obesity	Type 1 diabetes	1	10.61387687	1.11067E-06	1.03E-15	1203.089303	0.196441279
Atopic dermatitis	Alopecia areata	17	0.236851923	1.987619452	1.249455552	3.161882054	0.003728256
Alcoholic drinks per week	Alopecia areata	24	1.051683334	0.439028893	0.055883317	3.449086047	0.433782355
Smoking initiation	Alopecia areata	78	0.404381652	0.701639455	0.317612357	1.549996131	0.380899274
Obesity	Alopecia areata	1	21.72240247	9.2067E-10	2.97592E-28	2848302077	0.338158909

Atopic dermatitis	Systemic lupus erythema	15	0.134462169	1.059150591	0.813769117	1.378523652	0.669098152
Alcoholic drinks per week	Systemic lupus erythema	17	0.47988996	2.462443917	0.961337242	6.307495206	0.060403696
Smoking initiation	Systemic lupus erythema	58	0.236796288	0.834384809	0.524567426	1.327184981	0.444494325
Obesity	Systemic lupus erythema	1	12.73473626	107.1901219	1.54927E-09	7.41619E+12	0.713563029
Atopic dermatitis	Ankylosing spondylitis	17	0.122079967	1.118014626	0.880097768	1.420247555	0.36083149
Alcoholic drinks per week	Ankylosing spondylitis	24	0.542201931	1.155691398	0.399310086	3.344825625	0.789567341
Smoking initiation	Ankylosing spondylitis	78	0.208489656	1.402844685	0.932265502	2.110957885	0.104462959
Obesity	Ankylosing spondylitis	1	11.19614452	0.02420479	7.13758E-12	82082758.26	0.739613789
Atopic dermatitis	Vitiligo	2	0.705200188	0.481801719	0.120945531	1.919317681	0.300443525
Alcoholic drinks per week	Vitiligo	3	2.962455869	2.355014528	0.007084354	782.8651042	0.772478789
Smoking initiation	Vitiligo	8	1.55739962	2.125967662	0.10043182	45.00305262	0.628182802
Obesity	Vitiligo	0	92.20228304	6.69728E-11	2.1965E-89	2.04205E+68	0.799433929

Table S7. The results of MR-Egger intercept test

Exposure	Outcome	Egger_intercept	SE	P-value
Atopic dermatitis	Rheumatoid arthritis	0.019284157	0.037671561	0.615712121
Atopic dermatitis	Type 1 diabetes	0.030818449	0.035319505	0.395808201
Atopic dermatitis	Alopecia areata	0.061506021	0.072710517	0.409356304
Atopic dermatitis	Systemic lupus erythematosus	-0.075043176	0.075243524	0.332586017
Atopic dermatitis	Vitiligo	0.009783323	0.045981589	0.834039842
Atopic dermatitis	Ankylosing spondylitis	0.0037063	0.044316378	0.934325365
Rheumatoid arthritis	Atopic dermatitis	0.007499636	0.004146943	0.074595404
Type 1 diabetes	Atopic dermatitis	0.007825455	0.006430458	0.234559965
Alopecia areata	Atopic dermatitis	0.00185337	0.008620467	0.834562833
Systemic lupus erythematosus	Atopic dermatitis	-0.003583418	0.006643479	0.592685011
Vitiligo	Atopic dermatitis	-0.008285756	0.011831101	0.488940145
Ankylosing spondylitis	Atopic dermatitis	-0.0111109	0.01976907	0.59162632

SE: standard error

Table S8. Results of MR-PRESSO analysis in bidirectional MR analysis

Exposure	Outcome	MR.Analysis	OR	SD	P-value
Atopic dermatits	Rheumatoid arthritis	Raw	1.242158755	0.08765048	0.022441573
Atopic dermatits	Rheumatoid arthritis	Outlier-corrected	1.212178438	0.077668959	0.025621179
Atopic dermatits	Type 1 diabetes	Raw	1.421746887	0.140026626	0.02065193
Atopic dermatits	Type 1 diabetes	Outlier-corrected	1.19859355	0.07061398	0.020065842
Atopic dermatits	Alopecia areata	Raw	1.839656601	0.200965396	0.006321939
Atopic dermatits	Alopecia areata	Outlier-corrected	NA	NA	NA
Atopic dermatits	Systemic lupus erythematosus	Raw	1.359490671	0.225817859	0.188967386
Atopic dermatits	Systemic lupus erythematosus	Outlier-corrected	1.06152081	0.142435494	0.67980331
Atopic dermatits	Vitiligo	Raw	1.021296621	0.18586164	0.910859583
Atopic dermatits	Vitiligo	Outlier-corrected	0.861255294	0.137865729	0.292928733
Atopic dermatits	Ankylosing spondylitis	Raw	1.180507644	0.13160455	0.221162323
Atopic dermatits	Ankylosing spondylitis	Outlier-corrected	NA	NA	NA

Rheumatoid arthritis	Atopic dermatits	Raw	0.990459 291	0.015863 63	0.547307 541
Rheumatoid arthritis	Atopic dermatits	Outlier-corrected	0.999460 519	0.013162 419	0.967405 406
Type 1 diabetes	Atopic dermatits	Raw	0.992018 147	0.008846 131	0.370687 318
Type 1 diabetes	Atopic dermatits	Outlier-corrected	0.991294 975	0.006988 035	0.218946 852
Alopecia areata	Atopic dermatits	Raw	0.996860 081	0.007255 399	0.673064 956
Alopecia areata	Atopic dermatits	Outlier-corrected	NA	NA	NA
Systemic lupus erythematosus	Atopic dermatits	Raw	1.001491 822	0.008532 746	0.862151 21
Systemic lupus erythematosus	Atopic dermatits	Outlier-corrected	0.999623 168	0.008042 466	0.962849 02
Vitiligo	Atopic dermatits	Raw	0.973734 833	0.013282 467	0.052447 332
Vitiligo	Atopic dermatits	Outlier-corrected	0.972961 801	0.010330 565	0.012029 03
Ankylosing spondylitis	Atopic dermatits	Raw	0.992520 434	0.010667 954	0.495012 054
Ankylosing spondylitis	Atopic dermatits	Outlier-corrected	1.004865 863	0.010226 822	0.645238 526

OR,odds ratio ; SD, Standard Deviation
